# Process of Assay Selection and Optimization for the Study of Case and Control Samples from a Phase IIb Efficacy Trial of a Candidate Tuberculosis Vaccine, MVA85A

**DOI:** 10.1128/CVI.00128-14

**Published:** 2014-07

**Authors:** Stephanie A. Harris, Iman Satti, Magali Matsumiya, Lisa Stockdale, Agnieszka Chomka, Rachel Tanner, Matthew K. O'Shea, Zita-Rose Manjaly Thomas, Michele Tameris, Hassan Mahomed, Thomas J. Scriba, Willem A. Hanekom, Helen A. Fletcher, Helen McShane

**Affiliations:** a Jenner Institute, University of Oxford, Oxford, United Kingdom; b South African Tuberculosis Vaccine Initiative, Institute of Infectious Disease and Molecular Medicine and School of Child and Adolescent Health, University of Cape Town, Cape Town, South Africa; c Division of Community Health, Stellenbosch University, Cape Town, South Africa; d Metropolitan District Health Services, Western Cape Government, Cape Town, South Africa

## Abstract

The first phase IIb safety and efficacy trial of a new tuberculosis vaccine since that for BCG was completed in October 2012. BCG-vaccinated South African infants were randomized to receive modified vaccinia virus Ankara, expressing the Mycobacterium tuberculosis antigen 85A (MVA85A), or placebo. MVA85A did not significantly boost the protective effect of BCG. Cryopreserved samples provide a unique opportunity for investigating the correlates of the risk of tuberculosis disease in this population. Due to the limited amount of sample available from each infant, preliminary work was necessary to determine which assays and conditions give the most useful information. Peripheral blood mononuclear cells (PBMC) were stimulated with antigen 85A (Ag85A) and purified protein derivative from M. tuberculosis in an *ex vivo* gamma interferon (IFN-γ) enzyme-linked immunosorbent spot assay (ELISpot) and a Ki67 proliferation assay. The effects of a 2-h or overnight rest of thawed PBMC on ELISpot responses and cell populations were determined. Both the ELISpot and Ki67 assays detected differences between the MVA85A and placebo groups, and the results correlated well. The cell numbers and ELISpot responses decreased significantly after an overnight rest, and surface flow cytometry showed a significant loss of CD4^+^ and CD8^+^ T cells. Of the infants tested, 50% had a positive ELISpot response to a single pool of flu, Epstein-Barr virus (EBV), and cytomegalovirus (CMV) (FEC) peptides. This pilot work has been essential in determining the assays and conditions to be used in the correlate study. Moving forward, PBMC will be rested for 2 h before assay setup. The ELISpot assay, performed in duplicate, will be selected over the Ki67 assay, and further work is needed to evaluate the effect of high FEC responses on vaccine-induced immunity and susceptibility to tuberculosis disease.

## INTRODUCTION

Disease caused by Mycobacterium tuberculosis continues to be a major global health problem. In 2012, there were 8.6 million new cases of tuberculosis (TB) worldwide and 1.3 million people died of the disease (1). Bacille Calmette-Guérin (BCG), the only licensed TB vaccine, has variable efficacy, ranging from 0 to 80%, depending on the geographical location and population (2). A vaccine which is able to provide universal protection is urgently needed. The lack of a known correlate of protection against disease caused by infection with M. tuberculosis continues to be a major obstacle for the TB vaccine field, making it difficult to select which vaccines should progress to large-scale efficacy trials and to predict how successful those vaccines will be. Since 2002, more than a dozen candidate vaccines have entered into clinical testing (3), with the aim of boosting the efficacy of BCG or replacing it altogether. Only a few of these candidate vaccines have progressed to large-scale efficacy trials (3). The results of the most advanced of these, a phase IIb safety and efficacy trial of modified vaccinia virus Ankara, expressing the M. tuberculosis antigen 85A (MVA85A) in BCG-vaccinated South African infants, were published in February 2013 (5). MVA85A did not significantly improve the efficacy of BCG in this population, despite promising preclinical data from animal models (6−9) and the induction of potent and durable T cell responses in earlier phase I/IIa clinical trials (10–13). Although enhanced protection was not achieved in this population, peripheral blood mononuclear cells (PBMC) stored pre- and postvaccination provide a unique opportunity to investigate immunological differences between those infants who went on to develop TB disease and those who did not. With limited PBMC available to do this, careful planning was needed in order to select the assays which give the most relevant and diverse information. Prior work using the mycobacteria growth indicator tube (MGIT) assay and gene expression analysis demonstrated that high-quality data can be obtained using samples from the same population of South African infants, and these two assays (our unpublished data) will be included in the correlate analysis. The aim of this work was to determine which other assays have utility for inclusion. Here we describe some pilot work carried out to evaluate the optimum time that thawed PBMC should be rested before setting up immunological assays and compare antigen-specific responses to antigen 85A (Ag85A) and purified protein derivative (PPD) from M. tuberculosis in the gamma interferon (IFN-γ) enzyme-linked immunosorbent spot assay (ELISpot) and the Ki67 proliferation assays. The utility of cell surface flow cytometry was also evaluated. This process of assay selection and optimization, prior to the processing of the valuable correlate samples, has relevance for all trials of new vaccines, where the amount of sample available for analysis will always be limited.

## MATERIALS AND METHODS

### Origin of samples. (i) Infant samples.

Samples used in these pilot experiments were cryopreserved PBMC from a double-blind, randomized, placebo-controlled phase IIb efficacy trial of the candidate TB vaccine, MVA85A, in BCG-vaccinated, HIV-negative South African infants (South African National Clinical Trials Register DOH-27-0109-2654, ClinicalTrials.gov registration no. NCT00953927). Collection of these trial samples was approved by the University of Cape Town Faculty of Health Sciences Human Research Ethics Committee, the Oxford University Tropical Research Ethics Committee, and the Medicines Control Council of South Africa. The samples used were selected from a subgroup of infants on whom gene expression analysis had already been planned, and the remainder were from those infants who were lost to follow-up. They were not from cases (infants who went on to develop TB disease) nor from matched controls.

### (ii) Adult samples.

Samples used in this study were cryopreserved PBMC from BCG-vaccinated, United Kingdom adults, 28 days after MVA85A vaccination (1 × 10^8^ PFU) (ClinicalTrials.gov registration no. NCT01194180).

### Experimental design.

Infants enrolled in this trial were 4 to 6 months old and had received BCG (Danish 1331; Statens Serum Institute [SSI], Denmark) within 7 days of birth. They were randomized to receive an intradermal vaccination with either MVA85A (1 × 10^8^ PFU) or placebo (Candida skin test antigen) (5). Two subsets of samples were used in this work. The first set consisted of PBMC taken at 0 to 7 days prevaccination and 28 days postvaccination from 30 infants given MVA85A and 30 given placebo. These samples were used for the Ki67 proliferation assay, to quantify the proliferative potential of PBMC when stimulated with mycobacterial antigens, and for the *ex vivo* IFN-γ ELISpot assay, to quantify the magnitude of the response to mycobacterial and nonmycobacterial antigens. The ELISpot responses were also used to assess the intra-assay variation, comparing responses across either duplicate or triplicate antigen wells. The correlation between the two assays was investigated.

The second set of samples consisted of pre- and postvaccination PBMC from 15 infants from each group (MVA85A and Candida placebo). Due to the logistics of processing the large number of case and control samples in the TB correlates of risk study, it is desirable to thaw and rest PBMC overnight. This set of samples was used to compare *ex vivo* IFN-γ ELISpot responses when PBMC were rested for 2 h or overnight. A subset of 10 of these samples was used for cell surface flow cytometry to identify cell populations and determine differences between a 2-h and an overnight rest. As a comparison to the infant samples, PBMC from 12 adults at 28 days after MVA85A vaccination were subjected to the same thawing and resting conditions and were used for cell surface flow cytometry and IFN-γ ELISpot assays.

### Cell thawing.

Two vials of cryopreserved PBMC from each sample were rapidly thawed in a 37°C water bath and transferred to a 15-ml Falcon tube containing 10 ml R10 (RPMI medium, 10% fetal calf serum, 1% l-glutamine, 1% penicillin-streptomycin, 1% sodium pyruvate). PBMC were pelleted, supernatants were discarded, and the samples were resuspended in 10 ml R10 with 20 μl Benzonase (25 U/μl) (Merck Chemicals Ltd.) and rested overnight at 37°C and 5% CO_2_. For the 2-h versus overnight comparison, PBMC were split into equal volumes; one set was rested for 2 h, and one set was rested overnight. PBMC were counted on a Casy counter (Roche) and split into the appropriate volumes for each assay. In some cases, there were inadequate numbers of cells to perform all assays and use all conditions.

### *Ex vivo* IFN-γ ELISpot assay.

The *ex vivo* IFN-γ ELISpot assay was performed on both subsets of samples, thawed PBMC from prevaccination and 28 days postvaccination as previously described (13). In a 96-well ELISpot plate (Millipore), triplicate wells containing 3 × 10^5^ PBMC were stimulated with a single pool of Ag85A peptides, consisting of 66 15-mer peptides, overlapping by 10 amino acids (2 μg/ml/peptide) (Peptide Protein Research), BCG from pooled SSI vaccine vials (2 × 10^5^ CFU/ml), purified protein derivative (PPD) from M. tuberculosis (20 μg/ml) (SSI), FEC, consisting of a single pool of flu, Epstein-Barr virus (EBV), and cytomegalovirus (CMV) peptides (10 μg/ml/peptide) (Peptide Protein Research), and combined TB10.3 and TB10.4 peptides (10 μg/ml/peptide) (Peptide Protein Research). Phytohemagglutinin (PHA) (Sigma) was used as a positive control, and unstimulated wells were used as a measure of background IFN-γ production. The results are reported as spot-forming cells (SFC) per million PBMC, calculated by subtracting the mean of the unstimulated wells from the mean of triplicate antigen wells and correcting for the numbers of PBMC in the wells. A response was considered positive if the mean number of spots in the antigen well was at least twice the mean of the unstimulated wells and at least 5 spots greater.

### Ki67 proliferation assay.

PBMC were counted and plated in 24-well plates at 1 × 10^6^ PBMC/1 ml R10. Plates were incubated for 6 days at 37°C and 5% CO_2_ with either 2 μg/ml PPD or 1 μg/ml/peptide of a single pool of antigen 85A peptides. Two wells of PBMC were left unstimulated in R10. After 3 days, PHA was added to one of the unstimulated wells at 0.3 μg/ml. Three days later, the cells were washed in phosphate-buffered saline (PBS) and stained with amine-reactive viability dye (Live/Dead fixable red dead cell stain; Invitrogen). Cells were then permeabilized with Perm/Wash (BD Biosciences) and incubated with monoclonal antibodies: anti-CD3-AF700, anti-CD4-PB, anti-Ki67-phycoerythrin (PE) (BioLegend), and anti-CD8-allophycocyanin (APC)/AF750 (Beckman Coulter). Cells were then washed and acquired on a BD LSR II flow cytometer (BD Biosciences, San Jose, CA). Data were analyzed using FlowJo software (Tree Star Inc.). Dead cells were excluded from the analysis, while singlet CD3^+^ T cells, CD3^+^ CD4^+^ T cells, and CD3^+^ CD8^+^ T cells were included. Proliferating cells are presented as the percentage of Ki67^+^ T cells out of the total CD4^+^ or CD8^+^ T cells. Background (unstimulated) values were subtracted from all data.

### Cell surface flow cytometry.

PBMC were washed and stained with Live/Dead fixable red dead cell stain (Invitrogen) followed by surface staining with the following antibodies: anti-CD3-AF700, anti-CD4-PB, anti-CD14-PE/Cy7, anti-CD16-AF488, anti-CD19-PE/Cy5, anti-CD25-APC/Cy7, γδ T cell receptor (TCR)-APC, anti-cytotoxic-T-lymphocyte-associated antigen (CTLA)-PE (all from BioLegend), anti-CD8-efluorNC605, and anti-CD127-efluorNC650 (eBioscience). Fluorescence minus one (FMO) controls were used to identify boundaries of gates for CD25, γδTCR, CD127, and CTLA. Samples were acquired on a BD LSR II flow cytometer. Results are presented as percentages of cells after gating out of dead cells and doublets. CD4^+^ and CD8^+^ T cells were identified as CD3^+^ cells, while CD14^+/−^ and CD16^+/−^ cells were identified as CD3^−^ and CD19^−^ populations. CTLA^+^ and CD25^+^ CD27^−^ populations were gated on the CD4^+^ cells.

### Statistical analysis.

Statistical analyses were performed using GraphPad Prism and SPSS. The Mann-Whitney test was used to compare differences between groups. The Wilcoxon matched-pairs signed-rank test was used to compare responses after a 2-h or an overnight rest. Spearman's rho was used to determine correlations between the ELISpot and Ki67 proliferation assays. The intraclass correlation (ICC) with a two-way mixed-effects model was used to assess the reproducibility between duplicate and triplicate wells in the IFN-γ ELISpot assay.

## RESULTS

### Ki67 proliferation assay results correlate with IFN-γ ELISpot responses.

**FIG 1:**
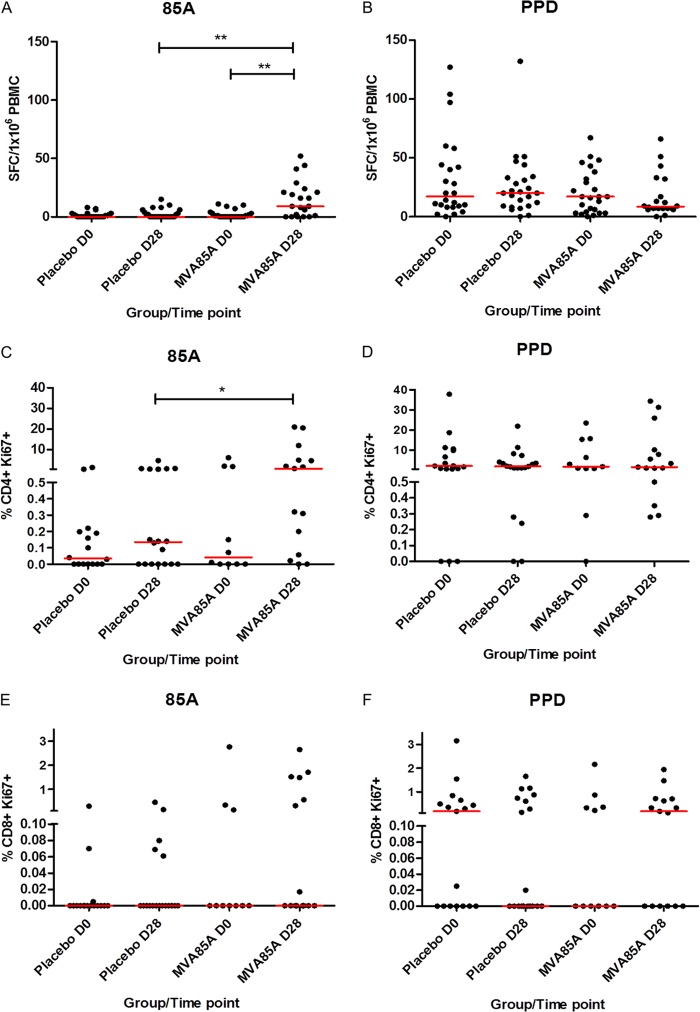
Detection of antigen-specific responses in infants vaccinated with MVA85A. IFN-γ ELISpot responses (A and B) and percentages of proliferating CD4^+^ Ki67^+^ (C and D) and CD8^+^ Ki67^+^ (E and F) in PBMC after stimulation with a single pool of antigen 85A peptides (A, C, and E) and purified protein derivative (PPD) from M. tuberculosis (B, D, and F). Red lines show median responses. Significant differences (Mann-Whitney test): *, *P* < 0.05; **, *P* < 0.01. *n* = 10 to 27 per time point.

Both the IFN-γ ELISpot and Ki67 assays detected significant differences (*P* = 0.001 and 0.03, respectively) between the MVA85A and placebo groups at 28 days postvaccination in response to stimulation with a single pool of antigen 85A peptides (Fig. 1A and C). A significant difference between the baseline and postvaccination responses in the MVA85A group was also detected using the ELISpot assay (*P* = 0.001) but was not detected in the Ki67 assay, possibly due to the low number of samples available for this assay in the MVA85A group on day 0 (D0). Neither assay detected any differences between groups or time points in response to stimulation with PPD (Fig. 1B, D, and F). Significant correlations were observed between the two assays for PPD responses at baseline and for both PPD and antigen 85A responses at D28 (vaccination groups combined) (Table 1).

**TABLE 1:** Correlations between percent CD4^+^ Ki67^+^ PBMC and IFN-γ ELISpot responses in South African infants after vaccination with either MVA85A or placebo (vaccination groups combined)

No. of days postvaccination	Antigen	Correlation coefficient (Spearman's rho)	*P*
0	85A	0.202	NS^*a*^
0	PPD	0.528	0.002
28	85A	0.494	0.005
28	PPD	0.362	0.042

a NS, not significant.

### Intra-assay variation.

The intraclass correlation (ICC) has been reported as an improved measure of reliability for quantitative data, where reliability is the reproducibility of a biological measurement when it is repeated for the same study subject (14). The ICC was used in this study to measure the reproducibility of responses detected in replicate wells of the ELISpot assay. The ICC improved as the magnitude of the detected IFN-γ ELISpot response increased. ICC values for duplicate (all combinations) and triplicate ELISpot wells indicated a good (ICC of 0.4 to 0.6) or very good (ICC of 0.6 to 0.8) level of agreement for baseline responses to antigen 85A and an excellent (ICC of 0.8 to 1.0) level of agreement for all other antigens and time points (Table 2).

**TABLE 2:** Intraclass correlation two-way mixed-effects model comparing reliability between duplicate and triplicate wells in the *ex vivo* IFN-γ ELISpot assay for 41 infant samples at baseline and D28

No. of days postvaccination	Antigen	ICC for^*a*^:			
		Wells 1 and 2	Wells 1 and 3	Wells 2 and 3	Wells 1, 2, and 3
0	85A	0.594	0.641	0.655	0.719
0	PPD	0.931	0.923	0.921	0.949
0	BCG	0.926	0.925	0.936	0.952
28	85A	0.934	0.904	0.895	0.939
28	PPD	0.858	0.855	0.834	0.895
28	BCG	0.892	0.868	0.896	0.920

a Intraclass correlation (ICC) values: 0 to 0.2, poor; 0.3 to 0.4, fair; 0.4 to 0.6, good; 0.6 to 0.8, very good; and 0.8 to 1.0, excellent.

### Cell counts are significantly lower overnight.

**FIG 2:**
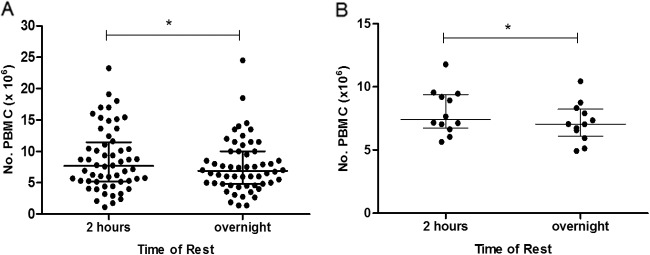
Cell viability is reduced in both adult and infant PBMC following overnight resting of cells. Infant (A) and adult (B) PBMC counts from a Casy counter (Roche) after a 2-h or overnight rest. Lines show median and interquartile ranges. An asterisk indicates significant difference (*P* < 0.05, Wilcoxon matched-pairs signed rank test). Infants, *n* = 56; adults, *n* = 12.

PBMC were counted on a Casy counter (Roche) after both the 2-h and overnight rests (Fig. 2). Infant 2-h counts ranged from a total of 1.1 × 10^6^ to 2.3 × 10^7^ cells, with overnight counts ranging from a total of 1.4 × 10^6^ to 2.5 × 10^7^ cells. A significant reduction in the median of the cell counts was observed, with counts of 7.7 × 10^6^ and 6.9 × 10^6^ for the 2-h and overnight rests, respectively (*P* = 0.02) (Fig. 2A). The median adult PBMC count also showed a significant reduction after the overnight rest (*P* = 0.03) (Fig. 2B).

### IFN-γ ELISpot responses are significantly lower after an overnight rest.

**FIG 3:**
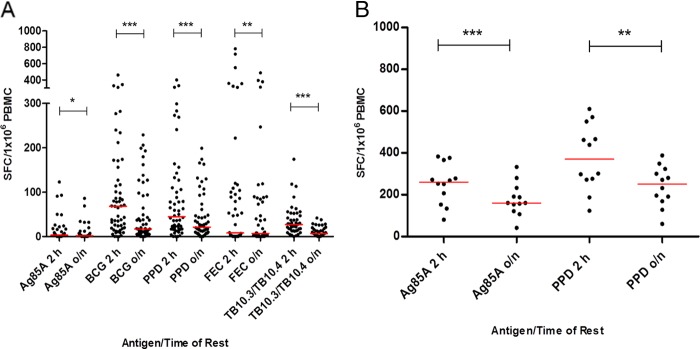
IFN-γ ELISpot responses are reduced in both adult and infant PBMC following overnight resting of cells. Infant (pre- and postvaccination) (A) and adult (postvaccination) (B) IFN-γ ELISpot responses after a 2-h or overnight rest. Red lines show median responses. Asterisks indicate significant differences (Wilcoxon matched-pairs signed-rank test): *, *P* < 0.05; **, *P* < 0.01; ***, *P* < 0.001. Infants, *n* = 45 to 55; adults, *n* = 12.

Figure 3 shows IFN-γ ELISpot responses after a 2-h and an overnight rest in both South African infants (groups and time points combined) and United Kingdom adults. Responses to all five antigens tested in the infant population were significantly lower after the overnight rest than after the 2-h rest (Wilcoxon matched-pairs signed-rank test), despite equivalent numbers of PBMC in the ELISpot wells (Fig. 3A). The same was true for the adult responses to the two antigens tested (Fig. 3B).

### Loss of T cell populations is significant after an overnight rest.

**FIG 4:**
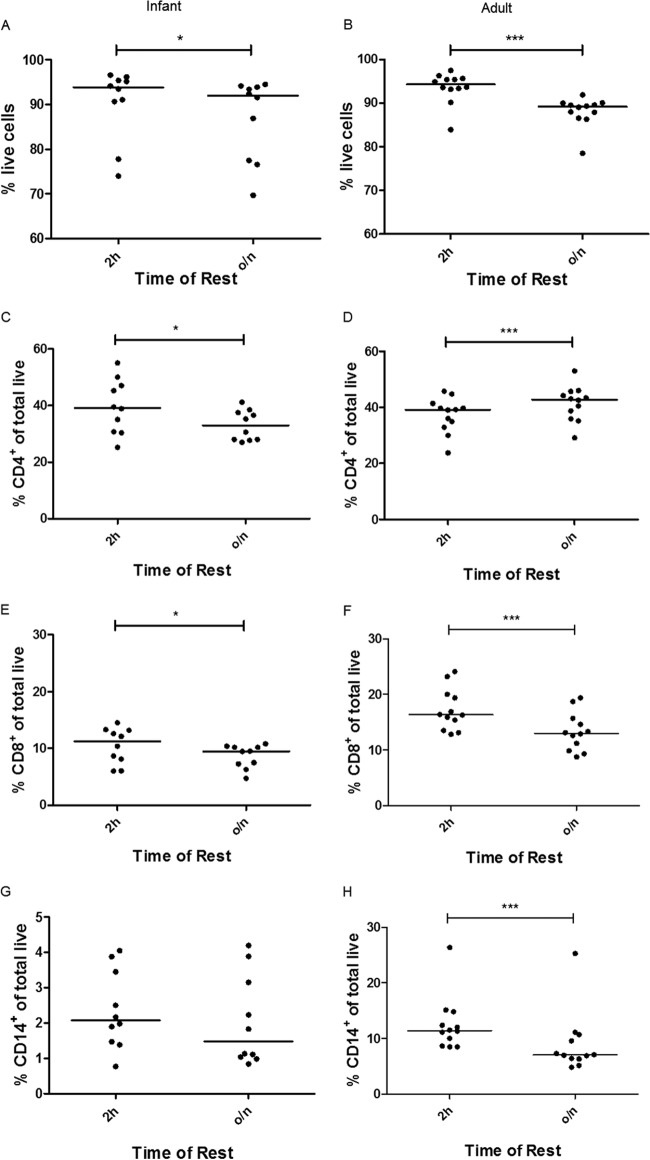
Changes in lymphocyte and monocyte cell frequencies following overnight resting of cells. Infant (left) and adult (right) cell viability (A and B) and percentages of total live CD4^+^ (C and D), CD8^+^ (E and F), and CD14^+^ (G and H) cells after a 2-h or overnight rest. Lines show median values. Asterisks indicate significant differences (Wilcoxon matched-pairs signed-rank test): *, *P* < 0.05; ***, *P* < 0.001. Infants, *n* = 10; adults, *n* = 12.

Cell surface flow cytometry of 10 infant samples and 12 adult samples showed a significant loss of viability overnight: a drop in median viability of 93.9 to 92.0% in the infants (*P* = 0.02) (Fig. 4A) and 94.3 to 89.2% in the adults (*P* = 0.0005) (Fig. 4B). The infant samples showed a significant loss of CD4^+^ and CD8^+^ T cells after an overnight rest compared to that after a 2-h rest (*P* = 0.01) (Fig. 4C and E) and a nonsignificant loss of CD14^+^ monocytes (Fig. 4G). In adults, a significant loss of CD8^+^ and CD14^+^ cells was observed (*P* = 0.0005) (Fig. 4F and H). While the percentage of CD4^+^ T cells significantly increased after an overnight rest (*P* = 0.0005) (Fig. 4D), the total number of CD4^+^ T cells did not.

### Proportion of infants responding on IFN-γ ELISpot assay to the FEC peptides was higher than expected.

A single pool of FEC peptides was included in the IFN-γ ELISpot assay for 30 infants. After a 2-h rest of PBMC, 15 out of 30 infants had a positive baseline or D28 response to these peptides, ranging from 27 to 783 SFC/million PBMC (median, 102 SFC/million PBMC) (Fig. 3A).

## DISCUSSION

Here we have presented preliminary work on the down-selection of assays and methods to be carried forward for investigating a correlate of infection/risk of tuberculosis. This work used pre- and postvaccination PBMC from a phase IIb efficacy trial of MVA85A in BCG-vaccinated South African infants and showed that high-quality data can be obtained from these cryopreserved samples.

Cell counts were variable, ranging from 1.1 to 23.3 million (median, 7.7 million) for the 2-h rest and dropping significantly overnight (median, 6.9 million). For most subjects, ample PBMC were available to carry out the desired assays and conditions, suggesting that it is worthwhile to plan to carry out multiple assays on the case versus control samples, as, based on the cell counts in this study, sample sizes will be large enough to make meaningful comparisons within each assay.

Both the *ex vivo* IFN-γ ELISpot and Ki67 proliferation assays were able to detect differences in responses to antigen 85A between the placebo and MVA85A-vaccinated groups at 28 days postvaccination. The ELISpot assay was also able to detect a difference between pre- and postvaccination responses to antigen 85A in the MVA85A group, which the Ki67 assay did not, possibly due to a lower number of samples available for the Ki67 assay in the MVA85A group at D0. The baseline responses to the PPD were high due to all infants receiving BCG vaccination at birth, but neither assay detected an increase in response to the PPD after vaccination with MVA85A. This is in contrast to previous findings in BCG-vaccinated United Kingdom adults and South African adults, adolescents, and children, where we see significant increases in responses to both Ag85A and PPD after vaccination with MVA85A (11, 13, 15). The lack of an increase in PPD responses in infants may be due to the recency of the BCG vaccination and the fact that the corresponding Ag85A responses are much lower than those we see in adults. There was good agreement between the two assays, with significant correlations for PPD responses at both the baseline and D28 and for antigen 85A responses at D28. The lack of correlation between the assays for antigen 85A responses at baseline is probably due to the very low responses detected at this time point. As a result of these findings and due to the cell-intensive nature and length of time involved in the Ki67 assay, we decided that the IFN-γ ELISpot assay would be taken forward for the full correlate analysis and the Ki67 proliferation assay would not be performed on further samples from this cohort.

Intra-assay variability was determined by looking at both duplicate and triplicate repeats in the ELISpot assay. Both numbers of repeats produced excellent ICC values, suggesting that it is sufficient to carry out the ELISpot with duplicate antigen wells, thus saving a considerable number of valuable PBMC for use in other assays.

Due to the logistical advantage, many trials of new vaccines perform analyses on cryopreserved PBMC. However, the loss of responses and therefore the loss of sensitivity of an assay with the use of cryopreserved samples instead of fresh must be minimized. Logistically, an overnight rest of thawed PBMC is desirable; however, a significant decrease in ELISpot responses for all of the antigens tested for both infant and adult samples was observed when PBMC were rested overnight compared with that when the same samples were rested for only 2 h. Cell surface flow cytometry to determine cell populations in the infant samples showed a significant loss of percentages of CD4^+^ and CD8^+^ T cells overnight, which might explain the loss of antigen-specific IFN-γ production in the ELISpot. A loss of monocytes, although not significant, might also result in lower levels of antigen presentation. As a consequence of this result, future assays will be set up after a rest of only 2 h as this will minimize the loss of antigen-specific responses.

The inclusion of FEC, a nonmycobacterial pool of CD8 epitopes, in the ELISpot assay provided some interesting results. While 50% of infants had no response to these peptides at either the baseline or D28, the remaining 50% had positive responses, with a median of 102 SFC/million PBMC. Infants in this trial were 4 to 6 months old when enrolled, so even at this very young age, a noticeable proportion have immunological memories of exposure to at least one of these viral pathogens. It will be of interest and possibly of relevance to determine which of these pathogens the infants are responding to. As a result, in the study of the correlate samples, separate pools of EBV, CMV, and influenza peptides will be included in the ELISpot panel.

An immune correlate analysis of the phase III HIV vaccine efficacy trial with RV144 used 6 assays for the primary analysis, selected on the basis of reproducibility, ability to detect postvaccine responses, and uniqueness of responses (16). The preliminary work we have shown here has also demonstrated the importance of selecting assays that are reproducible and sensitive enough to detect differences between groups and, importantly, those that measure different aspects of the immune response. This pilot study has been essential in determining the optimal assays and conditions to continue with for the TB immune correlate study.

## References

[1] World Health Organization. 2012 Global Tuberculosis Report 2012. World Health Organization, Geneva, Switzerland

[2] Colditz GA Brewer TF Berkey CS Burdick E Fineberg HV Mosteller F 1994 Efficacy of BCG vaccine in the prevention of tuberculosis. Meta-analysis of the published literature. JAMA 271:698–702 8309034

[3] Brennan MJ Thole J 2012 Tuberculosis vaccines: a strategic blueprint for the next decade. Tuberculosis 92:S6–S13. 10.1016/S1472-9792(12)70005-7 22441160

[4] Reference deleted

[5] Tameris MD Hatherill M Landry BS Scriba TJ Snowden MA Lockhart S Shea JE McClain JB Hussey GD Hanekom WA Mahomed H McShane H 2013 Safety and efficacy of MVA85A, a new tuberculosis vaccine, in infants previously vaccinated with BCG: a randomised, placebo-controlled phase 2b trial. Lancet 381:1021–1028. 10.1016/S0140-6736(13)60177-4 23391465 PMC5424647

[6] Vordermeier HM Villarreal-Ramos B Cockle PJ McAulay M Rhodes SG Thacker T Gilbert SC McShane H Hill AVS Xing Z Hewinson RG 2009 Viral booster vaccines improve Mycobacterium bovis BCG-induced protection against bovine tuberculosis. Infect. Immun. 77:3364–3373. 10.1128/IAI.00287-09 19487476 PMC2715681

[7] Verreck FA Vervenne RA Kondova I van Kralingen KW Remarque EJ Braskamp G van der Werff NM Kersbergen A Ottenhoff THM Heidt PJ Gilbert SC Gicquel B Hill AVS Martin C McShane H Thomas AW 2009 MVA.85A boosting of BCG and an attenuated, phoP deficient M. tuberculosis vaccine both show protective efficacy against tuberculosis in rhesus macaques. PLoS One 4:e5264. 10.1371/journal.pone.0005264 19367339 PMC2666807

[8] Goonetilleke NP Mcshane H Hannan CM Anderson RJ Brookes RH Hill AVS 2003 Enhanced immunogenicity and protective efficacy against Mycobacterium tuberculosis of bacille Calmette-Guérin vaccine using mucosal administration and boosting with a recombinant modified vaccinia virus Ankara. J. Immunol. 171:1602–1609. 10.4049/jimmunol.171.3.1602 12874255

[9] Williams A Goonetilleke NP Mcshane H Simon O Hatch G Gilbert SC Hill AVS Clark SO 2005 Boosting with poxviruses enhances Mycobacterium bovis BCG efficacy against tuberculosis in guinea pigs. Infect. Immun. 73:3814–3816. 10.1128/IAI.73.6.3814-3816.2005 15908420 PMC1111825

[10] Scriba TJ Tameris M Mansoor N Smit E van der Merwe L Mauff K Hughes EJ Moyo S Brittain N Lawrie A Mulenga H de Kock M Gelderbloem S Veldsman A Hatherill M Geldenhuys H Hill AVS Hussey GD Mahomed H Hanekom WA McShane H 2011 Dose-finding study of the novel tuberculosis vaccine, MVA85A, in healthy BCG-vaccinated infants. J. Infect. Dis. 203:1832–1843. 10.1093/infdis/jir195 21606542

[11] Hawkridge T Scriba TJ Gelderbloem S Smit E Tameris M Moyo S Lang T Veldsman A Hatherill M Merwe Van Der L Fletcher HA Mahomed H Hill AVS Hanekom WA Hussey GD McShane H 2008 Safety and immunogenicity of a new tuberculosis vaccine, MVA85A, in healthy adults in South Africa. J. Infect. Dis. 198:544–552. 10.1086/590185 18582195 PMC2822902

[12] Pathan AA Minassian AM Sander CR Rowland R Porter DW Poulton ID Hill AVS Fletcher HA McShane H 2012 Effect of vaccine dose on the safety and immunogenicity of a candidate TB vaccine, MVA85A, in BCG vaccinated UK adults. Vaccine 30:5616–5624. 10.1016/j.vaccine.2012.06.084 22789508 PMC3424417

[13] Meyer J Harris SA Satti I Poulton ID Poyntz HC Tanner R Rowland R Griffiths KL Fletcher HA McShane H 2013 Comparing the safety and immunogenicity of a candidate TB vaccine MVA85A administered by intramuscular and intradermal delivery. Vaccine 31:1026–1033. 10.1016/j.vaccine.2012.12.042 23266342 PMC5405058

[14] White E 2011 Measurement error in biomarkers: sources, assessment, and impact on studies. IARC Sci. Publ. 163:143–161 22997860

[15] Scriba TJ Tameris M Mansoor N Smit E van der Merwe L Isaacs F Keyser A Moyo S Brittain N Lawrie A Gelderbloem S Veldsman A Hatherill M Hawkridge A Hill AVS Hussey GD Mahomed H McShane H Hanekom WA 2010 Modified vaccinia Ankara-expressing Ag85A, a novel tuberculosis vaccine, is safe in adolescents and children, and induces polyfunctional CD4^+^ T cells. Eur. J. Immunol. 40:279–290. 10.1002/eji.200939754 20017188 PMC3044835

[16] Haynes BF Gilbert PB McElrath MJ Zolla-Pazner S Tomaras GD Alam SM Evans DT Montefiori DC Karnasuta C Sutthent R Liao HX DeVico AL Lewis GK Williams C Pinter A Fong Y Janes H DeCamp A Huang Y Rao M Billings E Karasavvas N Robb ML Ng J 2012 Immune-correlates analysis of an HIV-1 vaccine efficacy trial. N. Engl. J. Med. 366:1275–1286. 10.1056/NEJMoa1113425 22475592 PMC3371689

